# Supplementary material for: High-resolution live cell imaging to define ultrastructural and dynamic features of the halotolerant yeast Debaryomyces hansenii
Source: Biol Open. 2024 Jul 30;13(7):bio060519. doi: 10.1242/bio.060519 (PMC11317098; doi:10.1242/bio.060519)
Supplement: Supplementary information [file biolopen-13-060519-s1.pdf]

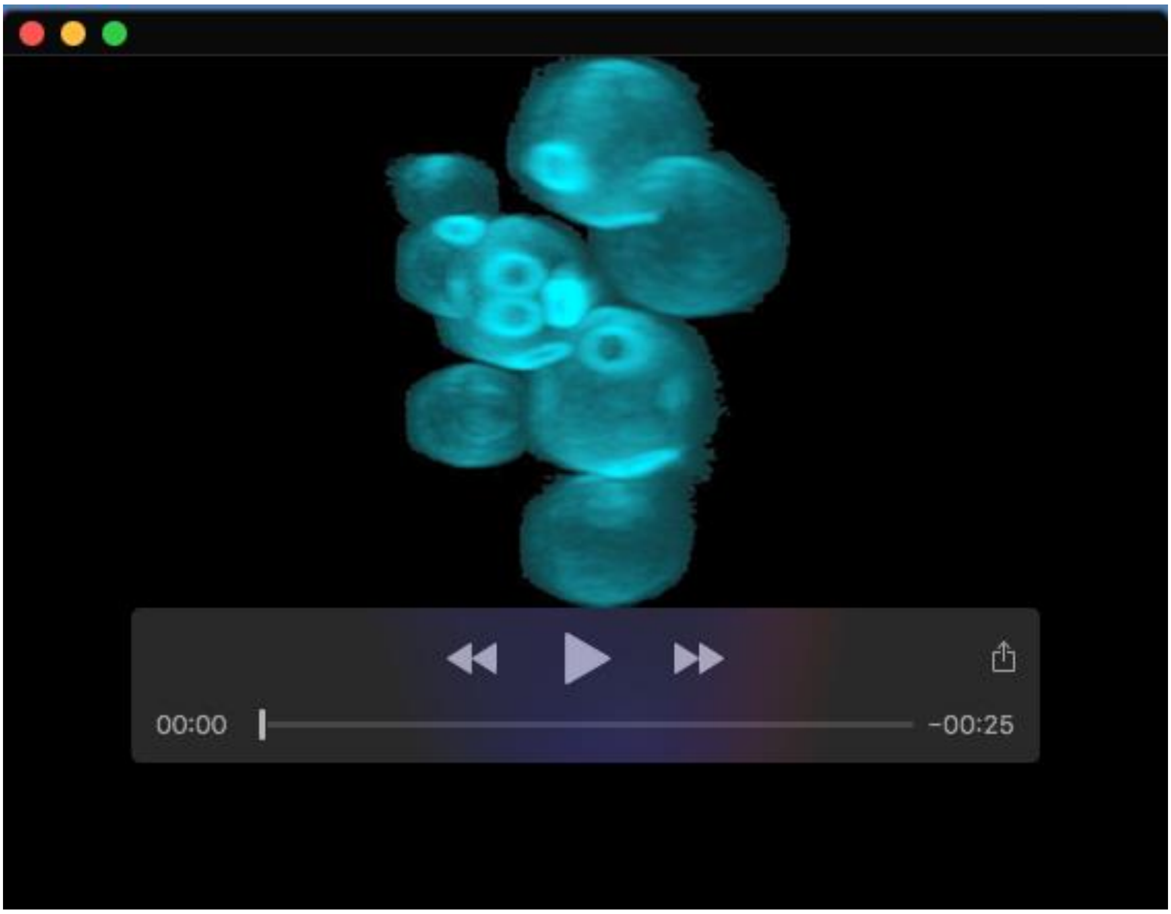

Movie 1. 3D volume of *D. hansenii* cell walls stained with Calcuflour white.
Log phase cells were stained with Calcuflour white in SC media for 5 minutes before washing and imaging in SC media. Airyscan2 confocal imaging was used to collect 45 individual z-stack slices, each 150nm. Zen blue was used to render a 3D projection across y-axis rotation and crop to indicated cells.

Biology Open • Supplementary information
